# The loss-of-function mutations and down-regulated expression of *ASB3* gene promote the growth and metastasis of colorectal cancer cells

**DOI:** 10.1186/s40880-017-0180-0

**Published:** 2017-01-14

**Authors:** Wu-Ying Du, Zhen-Hai Lu, Wen Ye, Xiang Fu, Yi Zhou, Chun-Mei Kuang, Jiang-Xue Wu, Zhi-Zhong Pan, Shuai Chen, Ran-Yi Liu, Wen-Lin Huang

**Affiliations:** 1 State Key Laboratory of Oncology in South China, Collaborative Innovation Center of Cancer Medicine, Sun Yat-sen University Cancer Center, Guangzhou, 510060 Guangdong P. R. China; 2 Department of Oncology, The First Affiliated Hospital, Sun Yat-sen University, Guangzhou, 510080 P. R. China; 3 Department of Radio-chemotherapy, Shangrao People’s Hospital, Shangrao, 334000 Jiangxi China; 4 Guangdong Provincial Key Laboratory of Tumor-targeted Drug and Guangzhou Enterprise Key Laboratory of Gene Medicine, Guangzhou Doublle Bioproducts Co. Ltd., Guangzhou, 510663 Guangdong P. R. China; 5 Department of Radiation Oncology, University of Arkansas for Medical Sciences, Little Rock, AR 72205 USA

**Keywords:** Ankyrin repeat and SOCS box protein 3 (ASB3), Colorectal cancer, Epithelial-mesenchymal transition, Cell proliferation, Tumor metastasis

## Abstract

**Background:**

Ankyrin repeat and SOCS box protein 3 (ASB3) is a member of ASB family and contains ankyrin repeat sequence and SOCS box domain. Previous studies indicated that it mediates the ubiquitination and degradation of tumor necrosis factor receptor 2 and is likely involved in inflammatory responses. However, its effects on oncogenesis are unclear. This study aimed to investigate the effects of ASB3 on the growth and metastasis of colorectal cancer (CRC).

**Methods:**

We used next-generation sequencing or Sanger sequencing to detect *ASB3* mutations in CRC specimens or cell lines, and used real-time quantitative polymerase chain reaction, Western blotting, and immunohistochemical or immunofluorescence assay to determine gene expression. We evaluated cell proliferation by MTT and colony formation assays, tested cell cycle distribution by flow cytometry, and assessed cell migration and invasion by transwell and wound healing assays. We also performed nude mouse experiments to evaluate tumorigenicity and hepatic metastasis potential of tumor cells.

**Results:**

We found that *ASB3* gene was frequently mutated (5.3%) and down-regulated (70.4%) in CRC cases. Knockdown of endogenous *ASB3* expression promoted CRC cell proliferation, migration, and invasion in vitro and facilitated tumorigenicity and hepatic metastasis in vivo. Conversely, the ectopic overexpression of wild-type *ASB3*, but not that of *ASB3* mutants that occurred in clinical CRC tissues, inhibited tumor growth and metastasis. Further analysis showed that ASB3 inhibited CRC metastasis likely by retarding epithelial-mesenchymal transition, which was characterized by the up-regulation of β-catenin and E-cadherin and the down-regulation of transcription factor 8, N-cadherin, and vimentin.

**Conclusion:**

*ASB3* dysfunction resulted from gene mutations or down-regulated expression frequently exists in CRC and likely plays a key role in the pathogenesis and progression of CRC.

## Background

In developed countries, colorectal cancer (CRC) is the third most common cancer in men and the second most common cancer in women [1, 2]. Although the incidence and mortality of CRC are lower in developing countries, including China, than in developed countries, they are rapidly rising with increasing economic development [1, 3]. Mounting evidences have confirmed that a series of gene mutations and epigenetic changes are involved in CRC tumorigenesis and progression [4–11]. Ubiquitination is a fundamental post-translational modification, and the ubiquitin–proteasome system plays an important role in regulating cell proliferation, apoptosis, angiogenesis, and motility. Additionally, abnormal regulations of the ubiquitin–proteasome system are known to promote colorectal carcinogenesis by regulating p53, Smad4, and components of the K-ras and Wnt/β-catenin pathways [12].

The ankyrin repeat and suppressor of cytokine signaling (SOCS) box (ASB) family contains 18 proteins, which interact with Cul5-Rbx2 to form a functional E3 ubiquitin ligase [13]. ASB proteins likely function as the substrate-recognition subunits of ElonginBC–Cullin–SOCS box (ECS)-type Cullin-Ring E3 ubiquitin ligase complexes that specifically transfer ubiquitin to cellular proteins for degradation by the proteasome [14]. ASB proteins, containing a SOCS box, are involved in the negative regulation of cytokine signaling [15]. Reportedly, ASB4 inhibits JNK activity and blocks insulin signal transduction by binding and inducing the ubiquitination and degradation of insulin receptor substrate 4 [16] and G-protein pathway suppressor 1 [17], as well as confers migration and invasion properties in hepatocellular carcinoma cells [18]. Furthermore, it was found that ASB9 expressed higher in CRC tissue than in corresponding normal tissue; that knockdown of ASB9 promoted the invasion of CRC cells; and that patients who expressed low levels of ASB9 had a lower overall survival rate than those who expressed high levels of ASB9 [19].

Human *ASB3* gene, another member of *ASB* gene family, is located on chromosome 2p16.2. It has three transcript variants that encode two isoforms. Isoform A of ASB3 contains 518 amino acid residues [20], which form 11 coterminous ankyrin (ANK) repeats followed by a SOCS box domain in the C terminal of the peptide [NCBI (The National Center for Biotechnology Information) Reference Sequence: NP_057199.1]. It has been reported that ASB3 mediates ubiquitination and degradation of tumor necrosis factor receptor 2, which plays a crucial role in several inflammatory responses [21]. In this study, we detected the mutations and expression of *ASB3* gene in CRC tissues and cells, and investigated the role of ASB3 in the pathogenesis of CRC.

## Methods

### Tissue samples

Paraffin-embedded and fresh frozen CRC specimens were collected from patients treated at Sun Yat-sen University Cancer Center, Guangzhou, China. All specimens contained matched cancer tissues (percentage of tumor cells ≥70%) and corresponding normal mucosal tissues (>5 cm laterally from the edge of the cancerous region). The study protocol was approved by the Institutional Review Board and the Human Ethics Committee of Sun Yat-sen University Cancer Center, and informed consent was obtained from each patient.

### Cell lines and cell culture

Human normal colon epithelium cell line FHC; human CRC cell lines HT-29, COLO205, LoVo, HCT116, SW620, SW480, and DLD-1; and the human embryonic kidney cell line 293T were obtained from the American Type Culture Collection. Human CRC cell line THC8307 was kindly provided by Prof. Rui-Hua Xu at Sun Yat-sen University Cancer Center [22]. The FHC cell line was cultured in Dulbecco’s Modified Eagle Medium (DMEM)/nutrient mixture F-12 media containing 100 ng/mL hydrocortisone, 10 ng/mL cholera toxin, 5 μg/mL insulin, and 5 μg/mL transferrin supplemented with 10% fetal bovine serum (FBS). COLO205 was cultured in RPMI-1640 medium supplemented with 10% FBS. All other cells were cultured in DMEM supplemented with 10% FBS. All materials for cell culture were from Invitrogen/ThermoFisher Scientific (Carlsbad, CA, USA).

### *ASB3* exonic sequence analysis

Genomic DNA was extracted from fresh frozen samples or cells using a Gentra Puregene Tissue Kit (Qiagen, Hilden, Germany). The exonic sequence was analyzed by next-generation sequencing at the Beijing Institute of Genomics, Chinese Academy of Sciences, Beijing, China. Sequencing files were deposited in the European Genome-phenome Archive under accession number EGAS00001001088. The exon sequence of the *ASB3* gene was analyzed by Sanger sequencing at Invitrogen Trading (Shanghai) Co. Ltd (Shanghai, China).

### Small interfering RNAs and transient transfection

*ASB3* small interfering RNAs (siRNAs) and negative control siRNA (sequences are shown in Table 1) were synthesized by Shanghai GenePharma Co. Ltd. (Shanghai, China). For transient transfection, THC8307 (2 × 10^5^/well) or SW620 cells (4 × 10^5^/well) were seeded in 6-well plates for 24 h and then transfected with siRNAs (100 pmol/well) using Lipofectamine 2000 (Invitrogen) according to the manufacturer’s instructions. The cells were cultured for 24 h in standard media and then used for further analysis at the indicated time points.

**Table 1:** The sequences of small interfering RNAs (siRNAs) involved in this study

Target	Name	Direction	Sequences
*ASB3*	siRNA1	Sense	5′-r(GGACUUGUUAAUACCACUU)dTdT-3′
		Antisense	5′-r(AAGUGGUAUUAACAAGUCC)dTdT-3′
	siRNA2	Sense	5′-r(GCAUUGACACCCUUAUCUU)dTdT-3′
		Antisense	5′-r(AAGAUAAGGGUGUCAAUGC)dTdT-3′
Negative control	siNC	Sense	5′-r(GCGACGAUCUGCCUAAGAU)dTdT-3′
		Antisense	5′-r(UAUGCUCAAGCUUUCUAGC)dTdT-3′

### Retroviral expression vector construction, packaging, and stable cell line construction

To generate retroviral expression vectors, the fragments of human wild type (WT) *ASB3* and the artificial loss-of-function *ASB3* mutant ΔSOCS [21] were amplified by polymerase chain reaction (PCR) from cDNA of THC8307 cells with specific primers (Table 2) and cloned into *Xho* I and *Not* I (or *Cla* I) sites of pLNCX2 plasmid (Clontech, Mountain View, CA, USA). *ASB3* mutant-expressing vectors were generated using the GENEART site-directed mutagenesis system (Invitrogen) based on *WT ASB3*-expressing vector with specific primers (Table 2). All constructs were verified by DNA sequencing analysis. The retroviruses were then packaged and tittered following the manufacturer’s instructions.

**Table 2:** The sequences of primers used in this study

Target	Direction	Sequences
For qPCR		
*ASB3*	Forward	5′-CATACTTATTTCATCGGGTGC-3′
	Reverse	5′-GGTAACTGCCAACTGTCCTC-3′
*GAPDH*	Forward	5′-CTCCTCCTGTTCGACAGTCAGC-3′
	Reverse	5′-CCCAATACGACCAAATCCGTT-3′
For vector construction (*italics* indicates restriction enzyme recognition sequence)		
WT *ASB3*	Forward	5′-GCG*CTCGAG*ATGGATTTTACAGAGGCT-3′
	Reverse	5′-GGC*GCGGCCGC*TTATCCATCTTGAATAGCTG-3′
ΔSOCS	Forward	5′-GCG*CTCGAG*ATGGATTTTACAGAGGCT-3′
	Reverse	5′-GGG*ATCGAT*TTACCTTTCAACAGCTGGTG-3′
For point mutation generation (capital letter indicates mutated nucleotide)		
G135E	Forward	5′- ggctgttgcttcaacacgAagcaaatgttaatggatc-3′
	Reverse	5′-gatccattaacatttgctTcgtgttgaagcaacagcc-3′
K339I	Forward	5′-gtgaacattcttttgaTatatggagcccagata-3′
	Reverse	5′-tatctgggctccatatAtcaaaagaatgttcac-3′
R362C	Forward	5′-cgagaagttttcgatatttTgctactttttgaggaaagg-3′
	Reverse	5′-cctttcctcaaaaagtagcAaaatatcgaaaacttctcg-3′

To construct stable *ASB3*-overexpressing cells, HCT116 or DLD-1 cells that endogenously expresses the mutated ASB3 at a low level were infected with each retrovirus with 8 μg/mL of polybrene (Sigma-Aldrich, Milwaukee, WI, USA) and then were selected with G418 (Calbiochem, La Jolla, CA, USA) for 2–3 weeks.

### Real-time quantitative PCR assay

Real-time quantitative PCR (qPCR) analysis was conducted as described previously [23]. Briefly, total RNA was extracted with Trizol Reagent (Invitrogen), reversely transcribed into cDNA with M-MLV reverse transcriptase (Promega, Madison, WI, USA), and sequentially subjected to qPCR analysis with the SYBR Green PCR Kit (Invitrogen) using primers shown in Table 2. The threshold cycle (Ct) values were determined and normalized against that of glyceraldehyde-3-phosphate dehydrogenase (GAPDH) internal control. The relative mRNA levels were shown as the value of 2^−ΔCt^ against the control group [24].

### Western blotting analysis

Western blotting was performed as described previously [24, 25]. Briefly, cell pellets were lysed in RIPA lysis buffer (Santa Cruz Biotechnology, Inc., Santa Cruz, CA, USA) followed by centrifugation to remove insoluble materials. Protein were then separated by using sodium dodecyl sulfate-polyacrylamide gel electrophoresis and transferred to polyvinylidene fluoride membrane. Blots were probed with specific primary antibody for ASB3 (1:250, Novus Biologicals, Littleton, CO, USA); E-cadherin, N-cadherin, vimentin, transcription factor 8 (TCF8), β-catenin, and zonula occludens-1 (ZO-1) (1:1000; Cell Signaling Technology, Inc., Beverly, MA, USA); and GAPDH (1:2000; Santa Cruz Biotechnology, Inc.), followed by reaction with horseradish peroxidase-conjugated secondary antibody. Signals were visualized using the enhanced chemiluminescent detection system (Amersham Biosciences, Piscataway, NJ, USA).

### Immunohistochemical assay

**Fig. 1:**
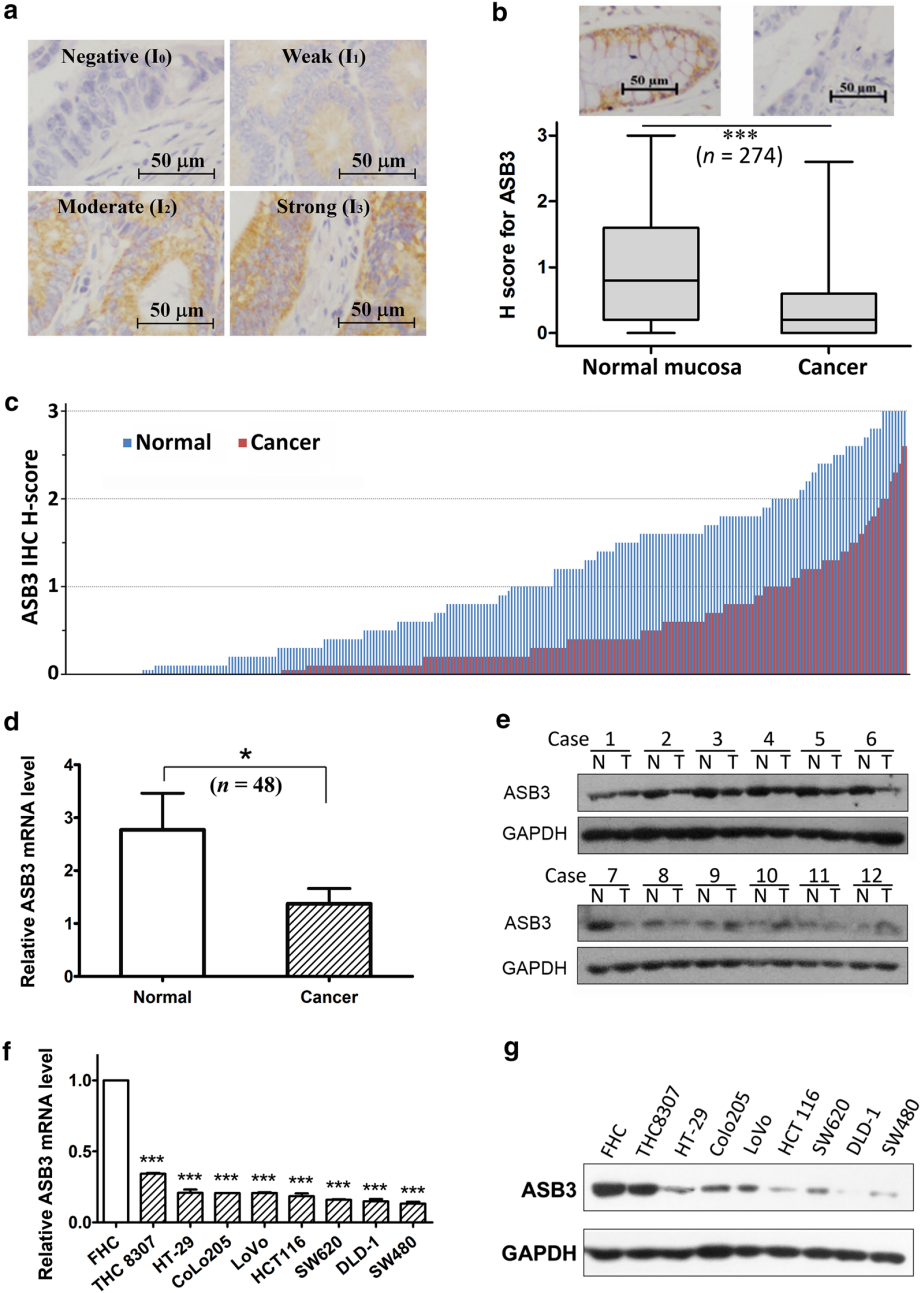
Ankyrin repeat and suppressor of cytokine signaling (SOCS) box protein 3 (ASB3) is generally down-regulated in colorectal cancer (CRC) tissues and cell lines. *ASB3* expression was detected by immunohistochemical (IHC), real-time quantitative polymerase chain reaction (qPCR), and Western blotting assays in CRC tissues (**a**, **b**, **c**, **d** and **e**) and cell lines (**f** and **g**). **a** Representative images of IHC staining for ASB3 in CRC tissues. ASB3 protein expression levels were divided into 4 levels: I_0_, negative; I_1_, weak staining; I_2_, moderate staining; I_3_, strong staining. The scale bar represents 50μm. **b** ASB3 protein levels (H score) determined by IHC assay in 274 pairs of CRC and corresponding normal mucosal tissues (***P < 0.001). **c** IHC H-scores ordered from low to high respectively. **d** The relative mRNA levels of ASB3 in 48 pairs of CRC and corresponding normal mucosal tissues (normalized against that of glyceraldehyde-3-phosphate dehydrogenase [GAPDH] internal control; *P = 0.016). **e** ASB3 protein levels detected by Western blotting assay in 12 pairs of CRC tissues (T) and corresponding normal mucosal tissues (N). GAPDH was used as a loading control. **f** *ASB3* mRNA levels detected by qPCR in normal intestinal epithelial cell line FHC and 8 CRC cell lines (***P < 0.001 compared with FHC cells). **g** ASB3 protein levels detected by Western blotting assay in cell lines; GAPDH was used as a loading control

Protein levels of ASB3 were detected by immunohistochemical (IHC) assay with a peroxidase kit (DAKO, Carpinteria, CA, USA) as described previously [26, 27]. Briefly, after routine deparaffinization, rehydration, and blocking with 0.3% H_2_O_2_ and antigen retrieval, the slides were incubated overnight at 4 °C with rabbit anti-ASB3 antibody (1:400, NBP1-88,812; Novus Biologicals), followed by incubation with HRP-conjugated secondary antibody and visualized with the EnVision Detection Kit (DAKO). Then, the sections were counterstained with hematoxylin. ASB3 staining intensity (I_0_, negative; I_1_, weak; I_2_, moderate; and I_3_, strong) (representative images shown in Fig. 1a) and the percentage of corresponding positive area (P_1_-P_3_) were evaluated by two pathologists who were blinded to clinical parameters. The ASB3 protein levels were presented as H score: H score = I_1_ × P_1_ + I_2_ × P_2_ + I_3_ × P_3_ [23, 28, 29].

### Cell proliferation assay

Cell proliferation was analyzed using MTT and colony formation assays as described previously [23, 25, 30]. For the MTT assay, cells were seeded in 96-well plates with a density of 2000 cells/well and incubated for indicated times. Cells were stained with MTT, and then absorbance was determined at 490 nm. For the colony formation assay, cells were seeded in 6-well plates at 500 cells/well and maintained in standard media for 14 days. Colonies were fixed with 4% paraformaldehyde and stained with crystal violet, and the ones containing more than 50 cells were counted.

### Cell cycle analysis

Cells were collected by centrifugation (1000 rpm × 10 min) after trypsinization, washed with ice-cold PBS twice, and fixed in 70% ethanol at 4 °C overnight. Cell suspensions were washed and re-suspended in PBS, treated with RNase A, and stained with propidium iodide. Finally, flow cytometry was performed to analyze cell cycle distribution [31].

### Wound healing assays

After serum deprivation for 12 h, confluent monolayers were scratched using a 10-μL pipette tip and washed once with serum-free medium to create a cell-free gap. Then, cells were incubated in DMEM medium containing 10% FBS. Wound healing in the same field was monitored and photographed under a microscope every 6 h from 0 to 36 h post-scratch [32, 33]. Using the Image-J software, images were then analyzed and calculated [34] to determine the rate of cell migration.

### Migration and invasion assays

Transwell assays were used to measure the migration or invasion ability of cells. In 200 μL of serum-free medium, 1 × 10^5^ cells were seeded into a Boyden chamber without or with Matrigel (8-μm pore; BD Falcon, San Jose, CA, USA) for migration or invasion assay, respectively; then the chambers were put in 24-well plates with 600 μL of medium containing 10% FBS. After 16–24 h of incubation, cells on the underside of the polycarbonate membrane were fixed in ethanol and stained with crystal violet; the numbers of migratory or invasive cells were determined from seven independent microscopic fields (200×).

### Immunofluorescence assays

Immunofluorescence assay was performed to detect the expression of epithelial-mesenchymal transition (EMT) markers. Cells cultured in glass-bottom cell culture dishes (NEST Biotechnology Co., Ltd., Wuxi, Jiangsu, China) were fixed with 4% paraformaldehyde, permeabilized with 0.5% Triton X-100, and blocked with 4% bovine serum albumin. Then, they were incubated with primary antibodies (Cell Signaling Technology, Inc.) for E-cadherin (1:100), vimentin (1:200), β-catenin (1:200), or N-cadherin (1:100) overnight at 4 °C, followed by incubation with Alexa fluor-594-conjugated or Alexa fluor-488-conjugated secondary antibody (Invitrogen). The samples were co-stained with DAPI and imaged by confocal laser scanning microscopy (Olympus FV1000; Olympus, Tokyo, Japan) [27].

### In vivo tumorigenicity and hepatic metastasis assays

Female BABL/c nude mice (4–5 weeks old) were purchased from the Experimental Animal Center of Guangdong Province, Guangzhou, China. All animal studies were performed following the United States National Institutes of Health (NIH) animal use guidelines and the current Chinese regulations and standards on the use of laboratory animals. All animal procedures were approved by the Sun Yat-sen University Institutional Animal Care and Use Committee.

For the tumorigenicity assay, the mice were injected subcutaneously with 1 × 10^6^ HCT116 cells overexpressing WT ASB3, ASB3 mutants [ΔSOCS, G135E, K339I, or G135E/K339I (a natural mutant containing G135E and K339I)], or vector-transfected control cells in 100 μL PBS (eight mice per group). Xenograft formation was monitored, tumor size was measured every 4 days, and tumor volume (V) was calculated according to the following formula: V = 0.52 × width^2^ × length [31, 35, 36]. Four weeks after implantation, the mice were euthanized, and the tumors were removed, photographed, and weighed.

For the hepatic metastasis assay, mice were anesthetized and subjected to laparotomy. One million HCT116 cells overexpressing WT ASB3 or ASB3 mutants or control cells in 20 μL PBS were respectively injected into the distal tip of the spleen (8 mice per group). Six weeks after incubation, the mice were euthanized and the spleens and livers were removed for pathologic examination [26, 27].

### Statistical analysis

All in vitro experiments were performed at least three times; all in vivo experiments were performed twice. Statistical analysis was conducted using the SPSS version 16.0 software (SPSS Inc., Chicago, IL, USA). Differences were analyzed by one-way analysis of variance (ANOVA) or exact χ^2^ test. Metering data are presented as mean ± standard deviation (SD). *P* values < 0.05 were considered statistically significant.

## Results

### *ASB3* gene was frequently mutated in CRC tissues and cell lines

Using next-generation sequencing (for 50 cases) and Sanger sequencing assays (for 83 new cases and 3 cases with *ASB3* mutations found in next-generation sequencing assays), we analyzed the *ASB3* gene mutations in tumor tissues collected from 133 CRC patients treated at Sun Yat-sen University Cancer Center, and found that *ASB3* gene had a high frequency of somatic mutations (5.3%, 7/133). We also performed Sanger sequencing to analyze *ASB3* gene exons in the normal intestinal epithelial cell line FHC and in CRC cell lines HCT116, HT-29, SW480, SW620, DLD-1, THC8307, CoLo205, and LoVo. We found that the *ASB3* gene mutated in HCT116, HT-29, and DLD-1 cells but not in other cells. The mutation types included missense, nonsense, and splice site mutations; of these, only missense mutations K339I and G135E occurred in Chinese CRC patients (Table 3).

**Table 3:** *ASB3* gene mutations in colorectal cancer tissues and cell lines

Mutation genotype	Mutation type	Exon	Codon change	Amino acid change	Case ID/Cell line
Colorectal cancer tissues					
CDS 21C→G^a^	Nonsense	Exon 2	TA**C**→TA**G** ^b^	Y7stop	C56
chr2:53810098 G→C	Splice-site	Exon 3/Intron	–	–	C53
CDS 404G→R	Missense	Exon 4	G**G**A→G**A**A	G135E	S78, S64
CDS 1084C→Y	Missense	Exon 8	**C**GC→**T**GC	R362C	S35
CDS 1016A→W	Missense	Exon 8	A**A**A→A**T**A	K339I	S12, S61, S64
Colorectal cancer cell lines					
CDS 471A→T	Missense	Exon 5	GA**A**→GA**T**	E157D	HCT116
CDS 471A→W	Missense	Exon 5	GA**A**→GA**T**	E157D	HT-29
CDS 24G→R	Synonymous	Exon 2	GC**G**→GC**A**	Non	DLD-1
CDS 25G→K	Missense	Exon 2	**G**AC→**T**AC	D9Y	DLD-1
CDS 154T→Y	Missense	Exon 2	**T**AT→**C**AT	Y52H	DLD-1
CDS 922G→R	Missensee	Exon 7	**G**CC→**A**CC	A308T	DLD-1

ASB3, ankyrin repeat and suppressor of cytokine signaling (SOCS) box protein 3

The nucleotides in *bold* indicate the one mutated

^a^ *CDS* coding domain sequence; A, T, C, G, four types of nucleotides; R = A/G; Y = C/T; W = A/T; K = G/T

^b^amino acid codon

### *ASB3* was down-regulated in most CRC tissues and cell lines

We detected the expression of *ASB3* by using IHC, qPCR and Western blotting assays. IHC assay for 274 CRC samples showed that average ASB3 protein levels were significantly lower in tumor tissues than in paratumor mucosa (*P* < 0.001) and that 70.4% (193/274) of CRC cases exhibited a down-regulated expression in tumor tissues (Fig. 1b, c). qPCR and Western blotting assays showed that ASB3 mRNA levels were significantly lower in CRC tissues than in paired normal colorectal mucous epitheliums (n = 48, P = 0.016; Fig. 1d) and that ASB3 protein levels were lower in 75.0% (9/12) of tumor tissues than in paired normal epithelial tissues (Fig. 1e). The examination of *ASB3* expression in CRC cell lines revealed similar results: *ASB3* expression was decreased at both mRNA and protein levels in CRC cells compared with FHC cells (Fig. 1f, g). These data suggest that *ASB3* expression is down-regulated in CRC.

### ASB3 inhibited the proliferation of CRC cells in vitro

**Fig. 2:**
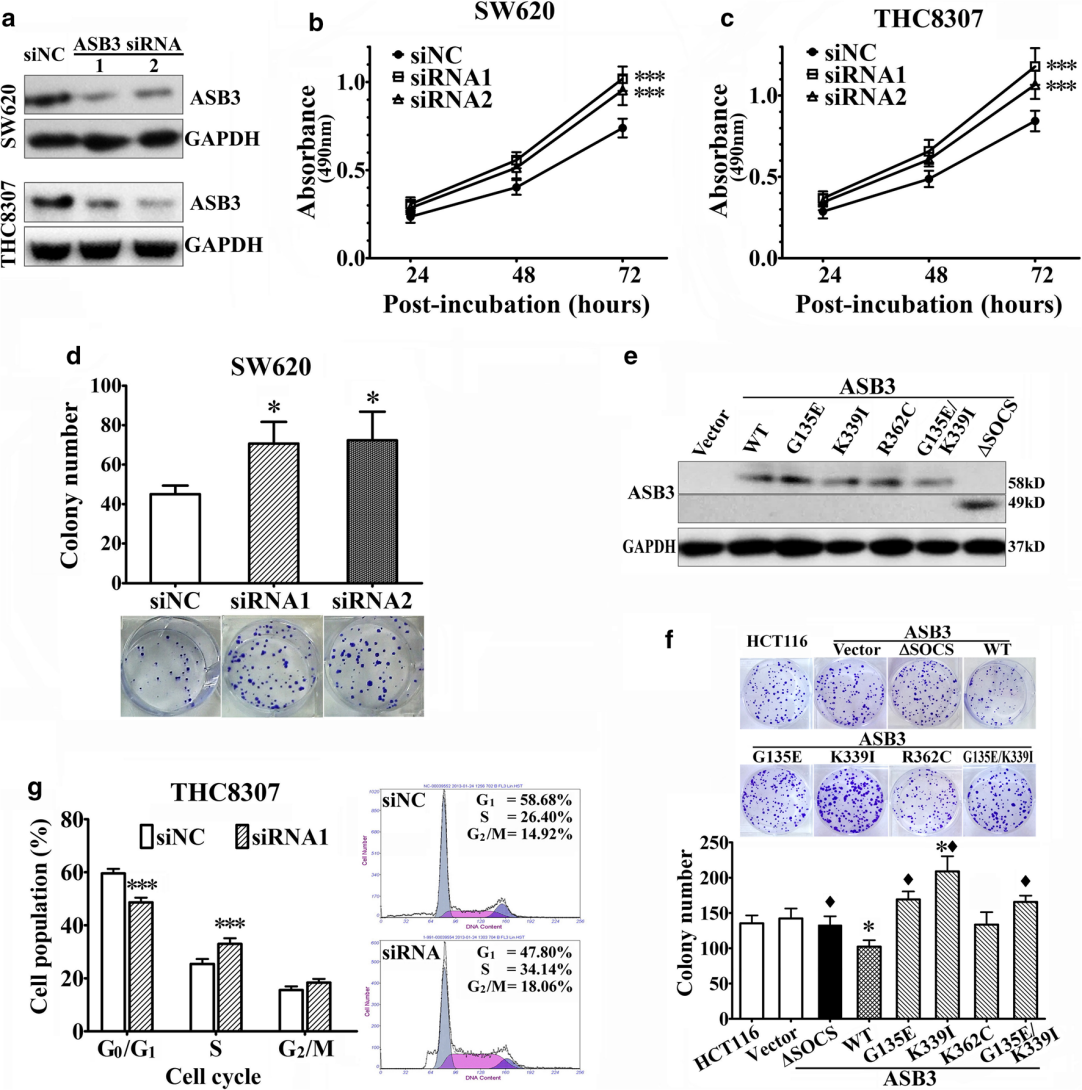
ASB3 inhibits the proliferation of CRC cells in vitro. ASB3 protein levels, cell proliferation, or cell cycle distributions were analyzed in CRC cells using Western blotting assay (**a** and **e**), MTT assay (**b** and **c**), colony formation assay (**d** and **f**), or flow cytometry (**g**) after ASB3 knockdown by transiently transfected with small interfering RNAs (siRNAs) or after ASB3 overexpression by stably transfected with *ASB3* cDNA or its mutants. siNC, negative control siRNA; WT, wild type; G135E, K339I, R362C, and ΔSOCS are ASB3 mutants. Representative pictures are also included in **d**, **f**, and **g**. *P < 0.05 compared with those transfected with siNC or vector control; ^♦^ *P* < 0.05 compared with that transfected with WT ASB3; ***P < 0.001 compared with that transfected with siNC

Since the *ASB3* gene is frequently mutated or down-regulated in CRC, we assumed that dysfunction of *ASB3* likely plays a role in the pathogenesis and progression of the disease. To confirm this hypothesis, first, we analyzed the effect of ASB3 on the proliferation of CRC cells in vitro. We performed *ASB3* knockdown in THC8307 and SW620 CRC cells that are with relatively high levels of WT ASB3 (Table 3; Figs. 1f, g, 2a) and then analyzed cell proliferation by MTT and colony formation assays. We found that the knockdown of *ASB3* significantly promoted CRC cell proliferation (Fig. 2b, c) and colony formation (Fig. 2d).

We performed colony formation assays in HCT116 CRC cells with a mutated *ASB3* gene which was expressed at a lower level (Fig. 1f, g; Table 3). We stably transfected *ASB3* cDNA or its mutants G135E, K339I, or R362C that were detected in clinical CRC tissues (Table 3) or ΔSOCS into HCT116 cells. The data showed that ectopic overexpression of WT *ASB3* inhibited HCT116 cell colony formation (Fig. 2e, f). However, overexpressing ASB3 mutants, including G135E, K339I, R362C, or ΔSOCS, did not inhibit HCT116 cell colony formation. On the contrary, K339I seemingly promoted colony formation (Fig. 2f). The inhibitory effects of these mutants (except R362C) on HCT116 cell colony formation were significantly different from that of WT ASB3 (Fig. 2f). This indicates that these mutants likely lose the inhibitory effect of WT ASB3 on CRC cell proliferation.

Next, we analyzed cell cycle distributions in THC8307 cells after *ASB3* knockdown and found that the down-regulation of *ASB3* expression promoted THC8307 cells from G_1_ into S phase of the cell cycle. In THC8307 cells transfected with *ASB3* siRNAs, the percentage of cells at G_1_ phase was significantly lower, and the percentage of cells at S phase was higher than those in control cells (*P* < 0.001, Fig. 2g). This indicates that *ASB3* inhibits CRC cell proliferation as a tumor suppressor and that dysfunctions of *ASB3* resulting from mutations or down-regulation are among the possible events that lead to CRC pathogenesis or progression.

### ASB3 inhibited the migration and invasion of CRC cells in vitro

**Fig. 3:**
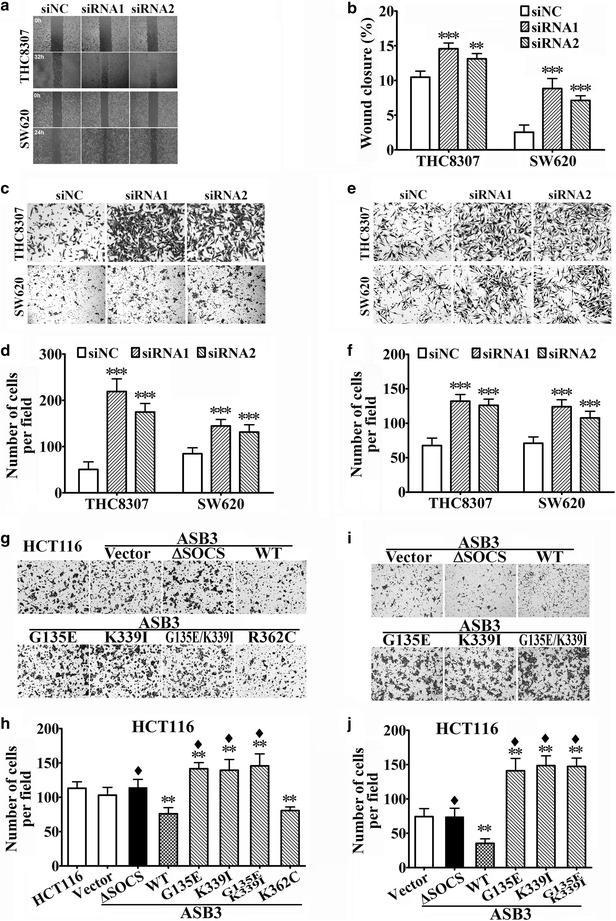
ASB3 inhibits the migration and invasion of CRC cells in vitro. The migration and invasion abilities of CRC cells were detected by wound healing and transwell assays after ASB3 knockdown by transiently transfected with siRNAs or overexpression by stably transfected with *ASB3* cDNA or its mutants. *siNC* negative control siRNA; *WT* wild type; G135E, K339I, R362C, and ΔSOCS are ASB3 mutants. **a** and **b** Wound healing assay, **c** and **d** transwell migration assay, and **e** and **f** invasion assay in THC8307 and SW620 cells after ASB3 knockdown. **g** and **h** transwell migration and **i** and **j** invasion assays in HCT116 cells after ASB3 (WT or mutants) overexpression. **P < 0.01, ***P < 0.001 compared with siNC or vector control; ^♦^ *P* < 0.01 compared with that transfected with WT *ASB3*

After artificially regulating *ASB3* expression in CRC cells as described above, we investigated the effects of ASB3 on cell migration and invasion in vitro. We found that *ASB3* knockdown facilitated wound healing, transwell migration, and invasion in THC8307 and SW620 CRC cells (Fig. 3a–f), whereas the overexpression of WT *ASB3* suppressed cell migration and invasion in HCT116 CRC cells (Fig. 3g–j). However, ASB3 mutants G135E, K339I, or G135E/K339I did not hinder but actually promoted HCT116 cell migration. Mutant ΔSOCS had no effect on cell migration, whereas mutant R362C acted similarly to WT ASB3 (Fig. 3g, h). The effects of ASB3 mutants G135E, K339I, G135E/K339I, and ΔSOCS (except R362C) on HCT116 cell migration were markedly different from that of WT ASB3 (Fig. 3h). Similar results were obtained from the invasion assay (Fig. 3i, j).

### ASB3 inhibited the tumorigenicity and hepatic metastasis of CRC cells in nude mice

**Fig. 4:**
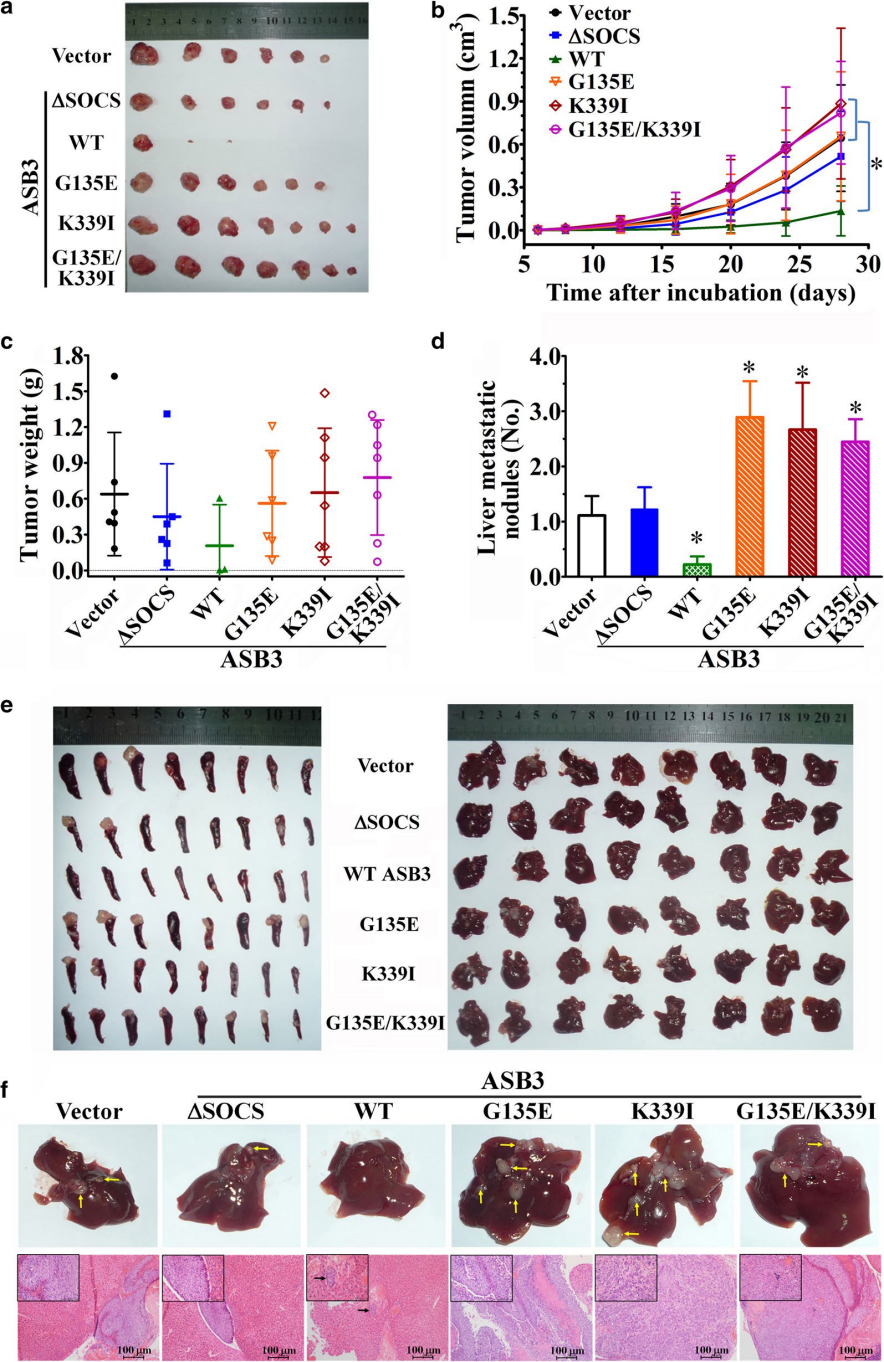
ASB3 inhibits the tumorigenicity and hepatic metastasis of CRC cells in vivo. HCT116 cells stably transfected with *ASB3* cDNA or its mutants (1 × 10^6^ cells each) were subcutaneously or intrasplenically injected into nude mice (8 in each group), and the formation and growth of subcutaneous xenografts **a**–**c** and liver metastatic tumors **d** and **e** were monitored. **a** The picture of subcutaneous xenografts. **b** Growth curves of subcutaneous xenografts (*P < 0.05). **c** Average weight of subcutaneous xenografts. **d** Numbers of liver metastatic tumor nodules (*P < 0.05 compared with the vector group). **e** Gross tumor growth in the spleen and liver after intrasplenic injection of HCT116 cells stably transfected with *ASB3* cDNA or its mutants (1 × 10^6^ cells each) for 8 weeks. *Left* general spleen images; *Right* general liver images. **f** Representative images (*upper*) and hematoxylin and eosin (HE) staining pictures (*lower*) of the livers from each group; the metastatic tumor nodules are indicated with *yellow arrows*. The *scale bars* in HE staining pictures represent 100 µm

To further elucidate the effects of ASB3 on the oncogenesis and progression of CRC, HCT116 cells stably overexpressing *ASB3* cDNA or its mutants (1 × 10^6^ cells each mouse) were subcutaneously or intrasplenically injected into nude mice, and the tumorigenicity and hepatic metastasis were investigated. We found that, in subcutaneous implant model, the tumor formation rates of subcutaneous xenografts of HCT116 cells overexpressing WT ASB3, ASB3 mutants G135E, K339I, G135E/K339I, and ΔSOCS were 37.5% (3/8), 75.0% (6/8), 87.5% (7/8), 87.5% (7/8), and 75.0% (6/8), respectively, whereas that of vector-transfected control cells was 75.0% (6/8) (Fig. 4a). The tumorigenicity of xenografts derived from HCT116 cells overexpressing WT ASB3 was lower, the volume of the tumors was smaller, and the weight of the tumors was lighter than those of xenografts from cells overexpressing ASB3 mutants or control cells (all *P* values < 0.05, Fig. 4b, c). In intrasplenic injection models, we found that overexpressing WT ASB3 in HCT116 cells suppressed intrasplenic tumor formation and hepatic metastasis (Table 4; Fig. 4d, e, f). However, overexpressing ASB3 mutants G135E, K339I, and G135E/K339I in turn facilitated hepatic metastasis (*P* < 0.05), whereas loss-of-function mutant ΔSOCS had no effects on hepatic metastasis (Table 4; Fig. 4d, e, f). These data indicate that overexpression of WT *ASB3* may suppress the tumorigenicity and hepatic metastasis of HCT116 cells in nude mice, whereas ASB3 mutants G135E, K339I, and G135E/K339I lose the inhibitory functions.

**Table 4:** The number and percentages of intrasplenic and hepatic tumor formation after intrasplenic injection of HCT116 stable cell lines (1×10^6^ cells each) for 8 weeks

Modified HCT116 stable cell line	Number of mice	Tumor formation^a^	
		Intrasplenic	Liver
Vectro	8	6 (75.0)	6 (75.0)
ΔSOCS	8	6 (75.0)	6 (75.0)
WT ASB3	8	4 (50.0)	2 (25.0)*
G135E	8	7 (87.5)	8 (100)
K339I	8	7 (87.5)	8 (100)
G135E/K339I	8	8 (100)	8 (100)

* *P* < 0.05 compared with other groups (Students’ *t* test)

^a^ The data are presented as the number of mice with tumors followed by percentage in parentheses

### ASB3 inhibited the EMT of CRC cells

**Fig. 5:**
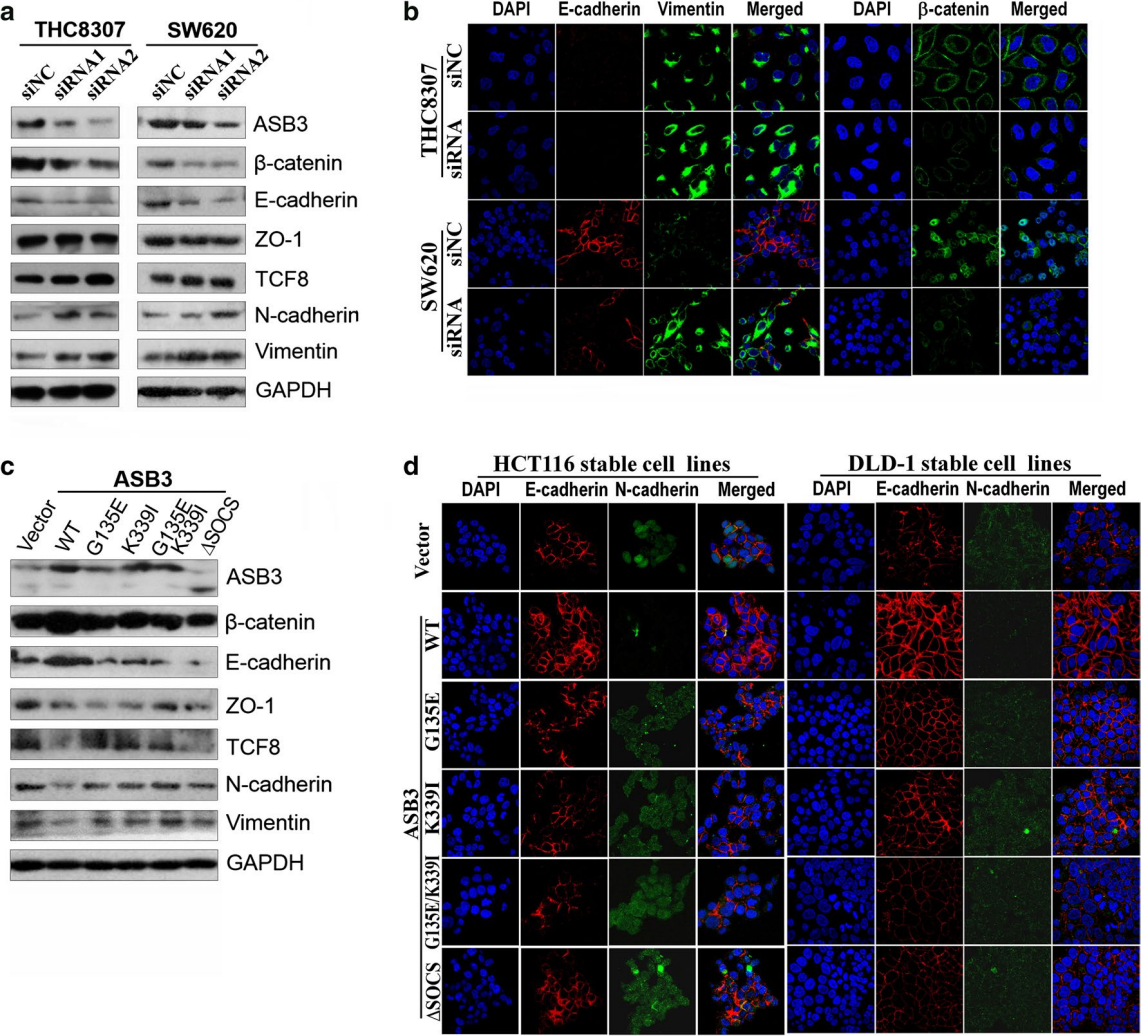
ASB3 inhibits the epithelial-mesenchymal transition (EMT) of CRC cells. After THC8307 and SW620 cells were transiently transfected with siRNAs to knockdown ASB3 expression for 48 h, EMT markers were detected with Western blotting (**a**) and immunofluorescence assays (**b**), whereas EMT markers were detected with Western blotting (**c**) and immunofluorescence assays (**d**) in HCT116 or DLD-1 cells stably transfected with *ASB3* cDNA or its mutants

Since ASB3 inhibited the metastasis of CRC cells in vitro and in vivo, we investigated whether ASB3 regulates EMT or not by Western blotting and immunofluorescence assays. The results showed that *ASB3* knockdown resulted in the down-regulation of β-catenin and epithelial marker E-cadherin and the up-regulation of TCF8 and mesenchymal markers N-cadherin and vimentin in THC8307 and SW620 CRC cells (Fig. 5a, b). Conversely, the ectopic overexpression of *ASB3* led to the up-regulation of β-catenin and E-cadherin and down-regulation of TCF8, N-cadherin, and vimentin (Fig. 5c, d). However, we found no obvious changes of these markers’ expression after overexpressing ASB3 mutants G135E, K339I, or G135E/K339I compared with the control (Fig. 5c, d).

## Discussion

ASB proteins were initially considered negative regulators of cytokine signaling because they contain SOCS box domain [15, 37]. However, mounting evidence has shown that ASB proteins are involved in many cellular processes and pathways. ASB11, an endoplasmic reticulum-associated ubiquitin ligase, interacts with and promotes the ubiquitination of ribophorin 1, which is involved in the glycosylation of nascent proteins [38]. ASB9 interacts with and promotes the ubiquitination and degradation of creatine kinase, and inhibits cell growth by negatively regulating mitochondrial energy metabolism [14, 39, 40]. ASB2α enhances adhesion of hematopoietic cells to fibronectin by degradating filamin A [41]. Furthermore, ASB2-involved ECS-type Cullin RING E3 ubiquitin ligase complex mediates mixed lineage leukemia (MLL) protein degradation during hematopoietic differentiation. One critical cause for MLL is likely that MLL fusion protein derived from chromosomal translocation is unable to be degraded due to the loss of the ASB2-binding site [42].

In the present study, we found that the *ASB3* gene had a high frequency of somatic mutations: it was mutated in 5.26% (7/133) of CRC cases and in HCT116, HT-29, and DLD-1 CRC cell lines. However, we observed that there are no obvious mutation hotspots in the *ASB3* gene in CRC (we consider that G135E and K339I are mutations reoccurred only in low frequency in CRC cases). Expression analysis showed that ASB3 was frequently down-regulated in CRC tissues and cell lines. Further investigations showed that the knockdown of *ASB3* promoted cell proliferation, migration, and invasion in cultured CRC cells, whereas the overexpression of WT *ASB3* inhibited cell proliferation, migration, and invasion in vitro and reduced tumorigenicity and hepatic metastasis of CRC xenografts in vivo. Furthermore, we found that overexpression of *ASB3* inhibited EMT of CRC cells, characterized by up-regulating epithelial markers β-catenin and E-cadherin and down-regulating mesenchymal markers TCF8, N-cadherin, and vimentin [43, 44]. Conclusively, ASB3 exerts a tumor-suppressive role in the pathogenesis and progression of CRC. However, the molecular mechanisms of how ASB3 regulates the proliferation, migration, and invasion remain unknown.

**Fig. 6:**
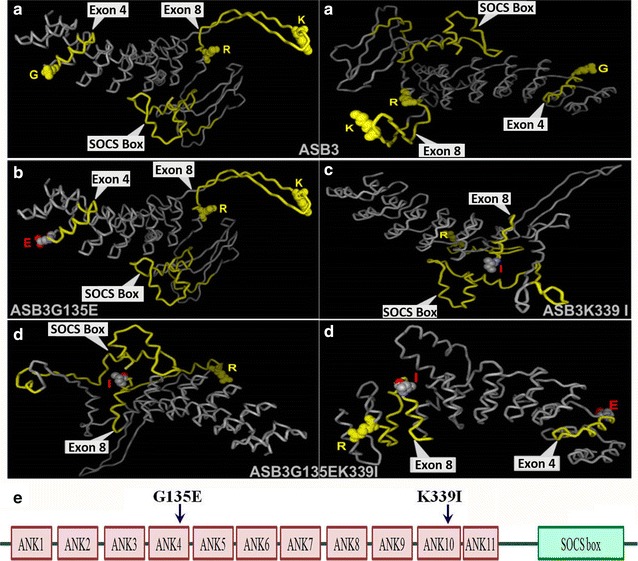
Comparison among three-dimensional (3D) models of WT ASB3 and ASB3 mutant proteins by computer homogeneity modeling and prediction with the SWISS-MODEL. The ASB3/ASB3 mutant proteins were modeled and predicted. **a** Images of spatial structure of ASB3 from two different angles. **b** Image of 3D model of G135E (ASB3G135E, on the* left*). **c** Image of 3D model of K339I (ASB3K339I, on the *right*). **d** Images of spatial structure of G135E/K339I (ASB3G135EK339I) from two different angles. **e** Structure sketch of ASB3 protein with G135E and K339I mutations

Like other ASB family members, ASB3 consists of an N-terminal ANK repeat domain (ARD) and a C-terminal SOCS box. Human ASB3 ARD contains 11 ANK repeats, called ANK1 to ANK11 (NCBI Reference Sequence: NP_057199.1), and is different from murine ASB3 that contains 12 ANK repeats [20, 21]. The ANK repeats are responsible for substrate recognition, whereas the SOCS box is responsible for assembling Elongin B/C, Cul5, and Rbx2 to form the E3 ubiquitin ligase complex [45]. Our current study demonstrated that not only ASB3 ΔSOCS mutant but also G135E, K339I, and G135E/K339I mutants that were detected in CRC cases lost the tumor-suppressive function of WT ASB3. Surprisingly, in some experiments, ASB3 mutants G135E, K339I, and G135E/K339I even displayed the opposite oncogenic effects. ASB3 mutant ΔSOCS loses its function resulting from the failure to recruit and compose E3 ubiquitin ligase complex. The ASB3 G135E mutation, encoded by exon 4, leads to an additional negative charge in ANK4 in physiological condition, whereas K339I, encoded by exon 8, leads to a loss of positive charge in ANK10. Theoretically, these two mutations will affect the spatial structure of ARD of ASB3, which is verified by the altered three-dimensional structure predicted by SWISS-MODEL (Fig. 6). Thus, we speculate that these ASB3 mutants affect substrate recognization, which makes ASB3 no longer recognize its original substrates, or even recognize novel substrates. The former case will result in a loss of the functions of WT ASB3, whereas the latter will bring in a novel function.

There are several limitations in our study. First, we did not clarify whether the *ASB3* dysfunction resulted from gene mutations or down-regulated expression affects the clinical prognosis for CRC cases due to the shorter follow-up. Second, further studies are required to confirm what molecules ASB3 directly interact with to play the tumor-suppressive role in CRC tumorigenesis.

## Conclusions

There are frequent mutations (5.3%) and down-regulated expression (70.4%) of the *ASB3* gene in Chinese patients with CRC. WT ASB3 inhibits CRC cell proliferation, migration, and invasion in vitro and decreases the tumorigenicity and hepatic metastasis in vivo; whereas mutated ASB3 lost this tumor-suppressive role. In conclusion, dysfunctions of the *ASB3* gene that result from mutations or down-regulated expression are possible events that lead to the pathogenesis or progression of CRC.

## Abbreviations

ANK: ankyrin
ARD: ANK repeat domain
ASB: ankyrin repeat and SOCS box protein
ANOVA: analysis of variance
CRC: colorectal cancer
EMT: epithelial-mesenchymal transition
FBS: fetal bovine serum
GAPDH: glyceraldehyde-3-phosphate dehydrogenase
h: hours
HRP: horseradish peroxidase
IHC: immunohistochemical
MTT: 3-(4,5-dimethyl-2-thiazolyl)-2,5-diphenyl-2-H-tetrazolium bromide
PBS: phosphate-buffered saline
qPCR: real-time quantitative PCR
SD: standard deviation
SDS-PAGE: sodium dodecyl sulfate-polyacrylamide gel electrophoresis
SOCS: suppressor of cytokine signaling
WB: western blotting
WT: wild type
